# A nation-wide cross-sectional study of variations in homecare nurses’ assessments of informational continuity – the importance of horizontal collaboration and municipal context

**DOI:** 10.1186/s12913-020-05313-3

**Published:** 2020-05-25

**Authors:** Marijke Veenstra, Marianne Sundlisæter Skinner, Maren Kristine Raknes Sogstad

**Affiliations:** 1 Norwegian Social Research, Oslo Metropolitan University, Oslo, Norway; 2 Centre for Care research, Norwegian University of Science and Technology (NTNU), Gjøvik, Norway

**Keywords:** Informational continuity, Continuity of patient care, Home nursing, Community health, Multilevel analyses, horizontal collaboration, Cross-sectional survey

## Abstract

**Background:**

Numerous studies have revealed challenges associated with ensuring informational continuity in municipal care services for older adults with comprehensive, prolonged and complex care needs. Most research is qualitative and on the micro-level. The aim of the current study is to map variation in homecare nurses’ assessments of available information in the municipalities’ documentation system and investigate the extent to which these assessments are associated with perceived quality of collaborations and with municipal context.

**Methods:**

We used data from a nationwide web-based survey among 1612 nurses working with older adults (65+) in homecare services in Norway. Responses from individual homecare nurses were linked with municipal-level data from the public registers. Data were analysed with descriptive statistics and multilevel regression analyses.

**Results:**

Information on the recipients’ medications and medical condition was considered most often available (42.8 and 20.0% responding very often/always), whereas information related to psychosocial needs and future follow-up was perceived less available (4.5 and 6.7% responding very often/always). Homecare nurses’ perceptions of the quality of collaboration with the GP and the allotment office were independently and positively associated with assessments of informational continuity (ß =0.86 and ß =0.96). A modest share of the total variation (8%) in assessments of informational continuity was at the structural level of municipality. Small municipalities (< 5000 inhabitants) had, on average, better informational continuity compared to larger municipalities (ß = -0.47).

**Conclusions:**

Documentation systems have a limited focus on long-term care needs of older care recipients beyond clinical and medical information. There is a potential for enhanced communication- and care-pathways between GPs, the allotment office and nurses in homecare services. This can support the coordinating role of homecare nurses in ensuring informational continuity for older adults with prolonged and complex care needs and help develop the facilitating role of (electronic) documentation systems.

## Background

The availability of patient information to providers throughout the healthcare system is crucial for patient safety and the quality of care services [1]. This applies particularly to older home dwelling adults with comprehensive and prolonged care needs [2]. In these instances, municipal health care services typically involve multiple care encounters over time with visits from more than one, often many, carer(s) [3]. Recent decentralization policies in Norway have contributed to a differentiation of municipal care services [4] with increased responsibilities for homecare nurses towards a higher number of older adults with complex care needs [5–8]. Unless relevant social, medical and nursing information is available and is transferred between carers and across care settings to connect care encounters, continuity of care is threatened [3, 9–11]. Informational continuity is one dimension of continuity of care [12] and refers to the use of information on past events and personal circumstances to make current care appropriate for the individual [13]. In order to maintain informational continuity, there is a need for systematic (electronic) written as well as oral information exchange between different providers in the municipal care setting, such as the primary physician (GP), the allotment office, persons working in short- and long-term institutional care and in homebased services [7, 11].

Extensive qualitative research has explored information practices within different municipal health care settings and has revealed a general lack of comprehensive nursing documentation [14–18]. This suggests that health care personnel has to make decisions based on incomplete information [18], which can lead to an incorrect allocation of services or even malpractice, with potentially serious consequences for care recipients. In addition, core providers in the Norwegian municipal health care sector must deal with different electronic systems for patient information. For example, GPs have different electronic systems than homecare nurses, and these systems do not communicate with each other. Instead, the GP has to use electronic messages (e-messages) to inform homecare services. The existence of such fragmented systems produces suboptimal opportunities for coordination as homecare nurses spend a disproportionate amount of time on documentation and on gathering necessary information from other health care providers or professions [19–23]. Nurses often depict this invisible organisation of work as inferior to direct patient contact, taking time away from care [20, 23]. However, if neglected, care recipients themselves, or their next of kin, have to keep track of information that is critical for their safety and quality of care [18, 24].

**Fig. 1:**
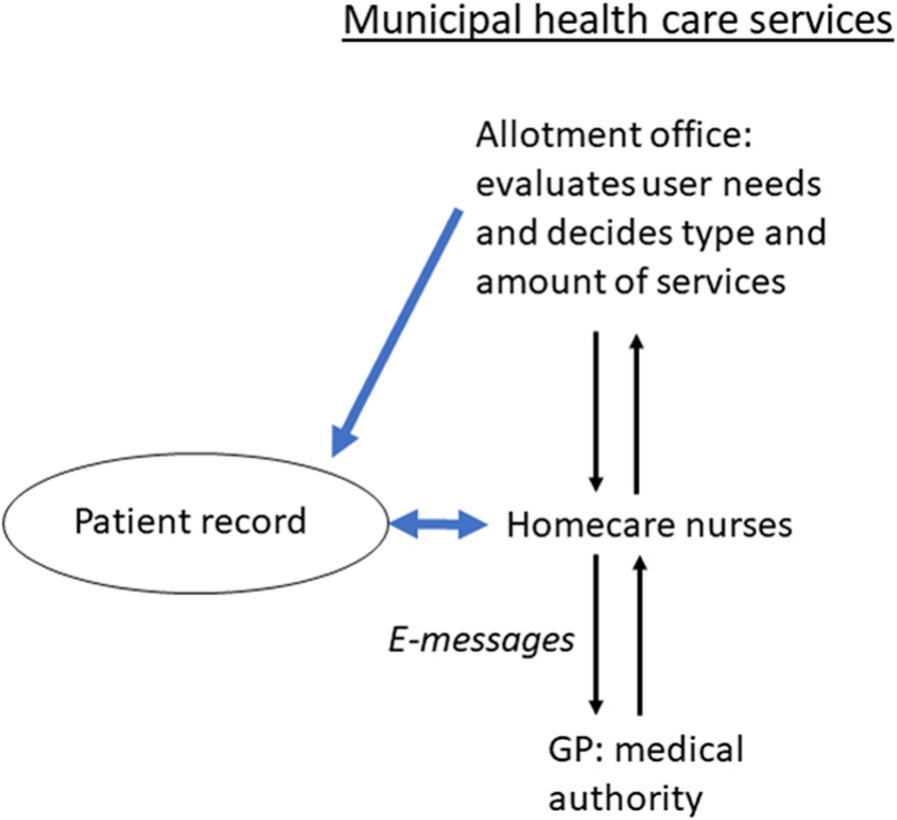
Overview of the information flow and horizontal collaborations between core providers within the Norwegian municipal health care system

Given the limitations of the (electronic) written documentation, collaboration between core providers within the municipal health care setting becomes increasingly important in ensuring informational continuity from one care episode to the next [25]. Horizontal collaboration is a general term indicating collaborations within a single organisational level of care (e.g. within the municipality) and a core strategy in municipal health care policies to ensure continuity of care [26, 27]. Figure 1 provides an overview of the information flow and collaborations between core providers in the Norwegian municipal health care system.

Studies have shown that different providers within the municipality collaborate in managing the fluctuating needs and everyday dilemmas of home based care [28, 29]. Whereas homecare nurses conduct the executive tasks within municipal homecare services, the administrative authority is in the hands of the municipality’s allotment office, who evaluates user needs and decides what type and amount of services shall be provided to specific users. The medical authority lies with the GP. Homecare nurses thus have limited ability to affect the allocation of care services or to change allocation decisions [7, 30]. Previous studies have highlighted the importance of these power differences for informational continuity [7]. Considering homecare nurses’ pivotal, and often invisible, role in ensuring informational continuity, more knowledge is needed of variations in homecare nurses’ perceptions of informational continuity and how these relate to collaborations with core providers. This would add to the predominantly qualitative observations in the field.

Ensuring high quality municipal health care, including appropriate and effective patient information systems, is the responsibility of the local authorities, in Norway as well as in many other countries [31]. This relatively autonomous role of municipalities has led to large variations in the organisation and prioritisation of care services between municipalities [32], and in particular in the allocation practices involving homecare services to older adults [6, 33, 34]. Homecare nurses working within the same municipality are therefore likely to have more similar assessments of informational continuity than nurses working in other municipalities. These similarities are further strengthened by the social construction of information practices [26, 27]. As most studies on informational continuity and documentation systems are qualitative, few studies have explored this interdependency and little is known about the potential role of structural conditions at the municipal level [35].

The current study aims to address these research gaps by (i) describing homecare nurses’ assessments of informational continuity; (ii) investigating the associations between informational continuity and collaboration with the allotment office and with the GP; and (iii) exploring the extent to which homecare nurses’ assessments of informational continuity are related to structural conditions at the municipal level. We hereby hypothesize that perceptions of better horizontal coordination between homecare nurses and the allotment office as well as between homecare nurses and the GP(s), are associated with more positive assessments of the availability of information. We also hypothesize that municipalities with more available resources (staffing, unrestricted revenues, and lower case-loads) have more positive assessments of information compared to municipalities with fewer available resources.

## Methods

We used data from a nationwide web-based survey among nurses working in municipal care services in Norway. Data were collected in 2017. Only nursing staff working in home care nursing or nursing homes and who were involved in the care of older adults (65 years and older) were included in the survey. A national register of nurses fitting our inclusion criteria does not exist, but the majority of Norwegian nurses are members of the Norwegian Nurses Organisation (NNO). The NNO granted access to the e-mail addresses from all 20,714 NNO members who were registered as working in the municipalities. Initial contact with the nurses was made by sending an e-mail containing information about the study and a link to the electronic questionnaire. The e-mail specified the inclusion criteria and asked only nurses who were part of the target group to participate. The e-mail also provided information concerning the aim of the study, the confidentiality of the data handling and the voluntary nature of participation. Informed consent was provided by returning the questionnaire confidentially. Screening questions identified nurses who did not fulfil the inclusion criteria. Three reminders were sent out: 1, 2 and 3 weeks after the initial e-mail contact. A total of 5884 nurses working in home nursing or nursing homes responded to the questionnaire and 5527 nurses indicated that their workplace received older patients (aged 65 years and older). Survey data has been de-identified, i.e. no personal identifiers are included. De-identified data are stored on a secure server at Oslo Metropolitan University. Only researchers involved in the research project have access to these secured data. The survey was reported to the Norwegian Centre for Research Data (NSD) who approved of the data collection and data storage.

### The questionnaire

The questionnaire built on questions tested in previous data collections on discharge planning [8, 36] and contained 32 questions on continuity in care services for older patients following hospital discharge. The median time it took to complete the questionnaire was 15 min. A first set of screening questions asked about the workplace and care setting, followed by questions about contacts with the hospital regarding the discharge of older patients, the quality of care transition and the quality of information transfer from the hospital to the municipality care setting. The second part contained questions about contacts between municipal care providers, the availability of information in the municipality’s documentation system, and the quality of care transition and collaborations within municipal care settings. The questionnaire finished with questions on background information, including gender, age (age group), working part-time (< 36 h per week), work experience and education.

### Sampling

In the current analyses, we selected respondents who were working in municipal homecare services (excluding those in nursing homes), who were involved in horizontal care transitions of older adults (65 years and older), and who had specified in which municipality they were working. The resulting analytic sample comprised 1612 homecare nurses of whom the majority were women (94%). Per January 2017, Norway counted 426 municipalities and 320 of these (75%) were represented in the current sample of homecare nurses. The smallest municipality had 506 inhabitants (in 2017), the largest municipality (the capital Oslo) had 673,468 inhabitants. The number of responding homecare nurses within each municipality ranged from 1 to 72.

### Dependent variable: availability of information

We used homecare nurses’ assessments of the availability of information about the care recipient in the municipality’s documentation system as an indicator of informational continuity. This was measured through the survey in the following manner: *When older patients are being transferred between municipal services, do you find sufficient information in the municipal documentation system about*: [1] the patients’ functional level [2]; the patients’ treatment plan [3]; the patients’ medical condition [4]; the follow-up received [5]; medications [6]; psychosocial needs; and [7] plans for future follow-up. Responses for each of the seven types of information were given on a five-point Likert scale, ranging from 1 (Never/Very seldom) to 5 (Very often/Always). These items were summated into a summary score ranging from 7 to 35, with higher scores indicating a higher degree of available information. Responses to a sixth response category “Not sure” were not included in the summary scores. This was the case for 0.2 to 1.4% across the seven items, resulting in 1421 nurses with valid responses on all items. Cronbach’s alpha of the resulting summated rating scale, labelled Availability of Information, is 0.84, indicating good reliability.

### Independent variables

Homecare nurses’ perceptions of collaboration with the allotment office and the GP were measured by asking: *Overall, how would you rate the collaboration with the following services in the municipality?* (i) The Allotment Office; and (ii) The GP. Responses to the two items ranged from 1 (Very good) to 5 (Very poor). We recoded the response categories so that higher scores indicated more positive experiences.

Data on the municipalities’ structural characteristics were derived from the national administrative database for Municipality-State-Reporting, KOSTRA, at Statistics Norway. All data refer to 2017, which corresponds to the year the survey among homecare nurses was conducted. We used the following variables to describe structural resources (staffing, financial means and caseload) at the municipal level: (i) Number of full-time equivalents (FTEs) per 10,000 inhabitants; (ii) unrestricted revenues in Norwegian Crowns (NOK) per 1000 inhabitants; (iii) the share of persons ≥80 years in the population, and the share of homecare recipients ≥80 years in the total ≥ 80 years population.

The unrestricted funds consist of the block grant and revenue from income tax and capital tax. There are relatively large variations in unrestricted revenues between municipalities in Norway, ranging from 34,000 to 151,000 NOK per inhabitant in 2017. In addition, we included demographic information about the municipality such as number of inhabitants (grouped into five categories: [1] Less than 5000 [2]; 5000–9999 [3]; 10,000-19,999 [4]; 20,000-49,999; and (5) 50,000 or more), and the centrality index based on travel distances for inhabitants to place of work and services. The original centrality index ranged from 0 (least central; i.e. almost no workplace or services within 90 min traveling) to 1000 (most central), and was categorized into six groups ranging from 1 (most central; i.e. Oslo and surroundings) to 6 (least central) [37].

### Potential confounders

Perceptions of horizontal collaborations as well of the availability of information may correlate with individual characteristics of home care nurses, including holding a management position, the number of years working at the current workplace and educational attainment. The level of post-qualifying education was measured as an ordinal variable with three categories: [1] No post-graduate education [2]; 1 year or less [3]; More than 1 year/master degree.

#### Statistical analyses

We used chi-square statistics (crosstabs) and analyses of variance to describe the data and variation across municipality groups (population size). Pearson correlations are used to summarize the bivariate associations in the data material, including between the independent variables, and act as a basis for the multivariate multilevel analyses and for the interpretation of the results. Only variables that showed statistically significant bivariate correlations with the dependent variable Availability of Information were included in the multivariate analyses. We conducted multilevel regression analyses [38] to analyse variation in nurses’ assessments across and within municipal contexts and to account for possible clustering of responses from nurses working within the same municipalities. Multilevel analyses allow for more efficient estimation of the statistical effects of the municipalities’ structural characteristics. We used empty random intercept models, without explanatory variables, to estimate the intraclass correlation coefficient (ICC). The ICC indicates the degree of clustering of homecare nurses’ responses within municipalities, and shows how much of the total variation in nurses’ assessments of information is at the municipal level. Random effects were included to test the extent to which the effects of collaboration with the GP and with the allotment office varied across municipalities. The random part of the multilevel model consists of variance in slopes between municipalities, variance in the intercept (overall mean given the explanatory variables are held at zero) and covariance between the slope and intercept. Variables with a significant random slope were centred to their grand mean. Significant improvements of subsequent models were assessed using a χ^2^–difference statistic related to the reduction in the Akaike Information Criterion (AIC). For each step, we also calculated the proportional reduction in prediction error [38], to approximate estimates of explained variance at the individual and municipal level. Another advantage of multilevel analysis is that municipalities with only one nurse also are included in the analyses. Eighty-two municipalities were represented with one homecare nurse. As a robustness check, we ran additional multilevel analyses including municipalities with five or more respondents, which gave similar findings (analyses available on request). For all statistical tests, we applied a 5% level of statistical significance (ɑ).

## Results

### Sample characteristics

Table 1 gives an overview of the sample characteristics of the 1612 nurses in the present study, and how these vary across municipality groups (population size). Almost half of the participants were younger than 40 years, had a postgraduate qualification (48%), and 43% had been working 5 years or less at their current workplace. Fifteen per cent reported having a management position. Working part-time was common, with 45% indicating that they worked less than 36 h per week. Participants from larger municipalities were younger, fewer were working part-time and had been working at their current workplace for a shorter time compared with small municipalities. Furthermore, Table 1 shows significant variation in the financial means, staffing and caseload across municipality groups (*p* < 0.001). For example, compared to the largest municipalities, municipalities with < 5000 inhabitants had higher unrestricted revenues per inhabitant (67,100 NOK versus 52,700 NOK), a higher number of FTEs (443 versus 280), and a larger share of people ≥80 years using homecare services (36% versus 28%).

**Table 1:** Sample descriptives homecare nurses (*N* = 1612) and municipalities (*N* = 320) included in the survey; across municipality groups (population size)

Population size	< 5000	5000–9999	10,000–19,999	20,000–49,999	> 49,999	Total
	**N (%)**	**N (%)**	**N (%)**	**N (%)**	**N (%)**	**N (%)**
***Characteristics homecare nurses***						
Age group***						
40 years and younger	110 (39.3)	96 (42.3)	116 (45.0)	196 (53.0)	254 (53.7)	772 (48.0)
41–50 years	89 (31.8)	58 (25.6)	78 (30.2)	96 (25.9)	117 (24.7)	438 (27.2)
51 years and older	81 (28.9)	73 (32.2)	64 (24.8)	78 (21.1)	102 (21.6)	398 (24.8)
Number of years at current work place***						
0–2 years	38 (13.7)	33 (14.6)	36 (14.0)	73 (19.7)	115 (24.5)	295 (18.4)
3–5 years	60 (21.6)	59 (26.1)	66 (25.7)	98 (26.5)	115 (24.5)	398 (24.9)
6–10 years	71 (25.5)	57 (25.2)	76 (29.6)	114 (30.8)	117 (24.9)	435 (27.2)
11–15 years	39 (14.0)	34 (15.0)	38 (14.8)	33 (8.9)	54 (11.5)	198 (12.4)
16–20 years	39 (14.0)	18 (8.0)	20 (7.8)	34 (9.2)	50 (10.7)	161 (10.1)
> 20 years	31 (11.2)	25 (11.1)	21 (8.2)	18 (4.9)	18 (3.8)	113 (7.1)
Missing (N)	2	1	1	0	4	8
Working parttime (% yes)*	141 (50.7)	99 (44)	121 (47.6)	167 (45.8)	185 (39.4)	713 (44.8)
Missing (N)	2	2	4	5	3	16
**Management position (% yes)**	43 (15.4)	40 (17.7)	40 (15.6)	50 (13.6)	72 (15.3)	245 (15.2)
Missing (N)	0	1	1	2	1	5
**Post-qualifying education**						
No	132 (47.3)	119 (53.1)	120 (47.1)	194 (52.9)	273 (58.1)	838 (52.5)
Yes −1 yr or less	89 (31.9)	62 (27.7)	73 (28.6)	108 (29.4)	116 (24.7)	448 (28.1)
Yes - more than 1 yr/masterdegree	58 (20.8)	43 (19.2)	62 (24.4)	65 (17.7)	81 (17.2)	309 (19.4)
Missing (N)	1	3	3	3	3	13
***Municipality characteristics***	Mean (SD)	Mean (SD)	Mean (SD)	Mean (SD)	Mean (SD)	Mean (SD)
Number of municipalities (N)	138	74	51	41	16	320
Number of inhabitants***	2670 (1189)	7017 (1417)	14,021 (2817)	29,218 (7648)	133,612 (156534)	15,433 (44431)
Centrality 0 (most)-1000 (least) (median)***	571	692	783	827	801	660
Share of persons aged ≥80 years (%) ***	5.9 (1.3)	4.9 (1.1)	4.1 (0.9)	4.1 (0.8)	3.9 (0.7)	5.1 (1.4)
Unrestricted revenues per inhabitant (in NOK) ***	67,100 (9400)	55,100 (4700)	52,700 (3600)	50,800 (3500)	52,700 (4200)	58,200 (9800)
Share of homecare recipients ≥80 years of the total 80+ population (%) ***	36.4 (6.4)	35.4 (5.7)	32.6 (4.3)	31.1 (4.6)	28.3 (2.6)	34.5 (6.1)
Number of FTEs in municipal health care services per 10,000 inhabitants***	443 (113)	356 (89)	300 (75)	295 (51)	280 (45)	372 (113)

Tests of statistical significance across municipality groups: *p < 0.05; ** p < 0.01; ***p < 0.001

### Availability of information

**Fig. 2:**
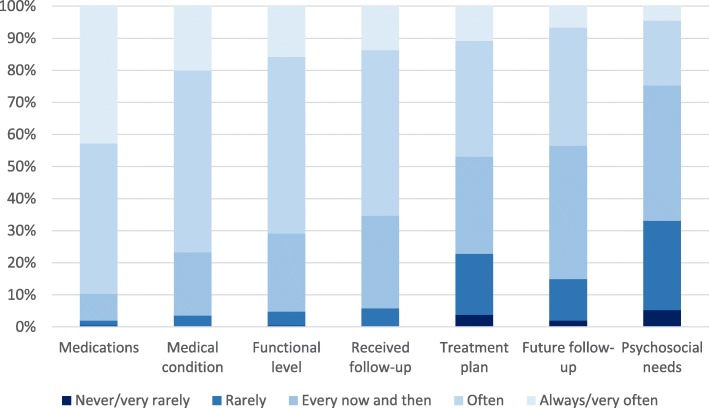
Response distribution (5-point scale) homecare nurses on the seven items measuring availability of information in the documentation system. *N* = 1421

Figure 2 shows the distribution of nurses’ responses on the seven single items measuring perceived availability of information. The share of homecare nurses’ reporting that information was Very often/Always available was highest for Medications (42.8%) and Medical Condition (20.0%) and lowest for Psychosocial Needs (4.5%) and Future Follow-up (6.7%).

Table 2 provides an overview of the percentage of nurses responding in the most positive response category for the single items measuring availability of information, and of the mean scores on the summated rating scale Availability of Information and the items measuring collaboration with the GP and the Allotment office. Differences across municipality groups were not statistically significant for the item measuring availability of information on the patient’s Treatment Plan. Homecare nurses ratings on the six other types of information differed significantly across municipality groups (*p* < 0.05). Mean scores on the summated rating scale for Availability of Information differed significantly across municipality groups as well (*p* < 0.001). Post-hoc multiple comparisons indicated that the two smallest municipality groups had significantly higher mean scores than the two largest municipality groups.

**Table 2:** Homecare nurses’ assessments of availability of information in the municipality’s documentation system (*N* = 1421) and collaboration with the Allotment office and the GP

Municipality size (population)	< 5000	5000-9999	10,000-19,999	20,000-49,999	> 49,999	Total
	N (%)	N (%)	N (%)	N (%)	N (%)	N (%)
**Types of Information Available** (% Info is available Always/Very often)						
Psychosocial needs**	20 (8.0)	8 (4.1)	11 (4.9)	6 (1.8)	19 (4.5)	64 (4.5)
Future follow-up**	27 (10.8)	10 (5.1)	15 (6.7)	17 (5.2)	26 (6.1)	95 (6.7)
Treatment plan	41 (16.4)	19 (9.6)	20 (8.9)	34 (10,4)	39 (9.2)	153 (10.8)
Received follow-up*	54 (21.6)	32 (16.2)	25 (11.2)	29 (8.9)	54 (12.7)	194 (13.7)
Functional level**	63 (25.2)	35 (17.8)	32 (14.3)	36 (11.0)	59 (13.9)	225 (15.8)
Medical condition**	74 (29.6)	49 (24.9)	42 (18.8)	42 (12.9)	77 (18.2)	284 (20.0)
Medications***	140 (56.0)	102 (51.8)	98 (43.8)	120 (36.8)	148 (34.9)	608 (42.8)
	**Mean (SD)**	**Mean (SD)**	**Mean (SD)**	**Mean (SD)**	**Mean (SD)**	**Mean (SD)**
Summated scale Availability of Information (7–35)***	26.26 (4.59)	26.07 (3.77)	25.33 (3.98)	24.87 (3.87)	24.8 (4.19)	25.33 (4.14)
**Collaboration: 1 (very poor) - 5 (very good)**						
^a^With Allotment office**	3.78 (0.89)	3.80 (0.93)	3.61 (0.96)	3.49 (0.97)	3.58 (0.96)	3.62 (0.95)
^b^With GP ***	3.92 (0.90)	3.76 (0.82)	3.72 (0.76)	3.49 (0.82)	3.54 (0.89)	3.65 (0.86)

Tests of statistical significance across municipality groups: *p < 0.05; ** p < 0.01; ***p < 0.001

^*a*^*N = 1301*

^*b*^*N = 1415*

Respectively 14.3 and 11.1% of the nurses assessed the collaboration with the Allotment Office and with the GP as “Very Good”. Homecare nurses’ mean score on both items was 3.6 (SD = 0.9) on a five-point scale, reflecting a tendency towards positive ratings.

Collaboration with the GP showed the highest bivariate correlation with information on Medications (r = 0.25) and the lowest correlation was with information on Treatment Plan (r = 0.13). Collaboration with the Allotment Office showed highest bivariate correlations with information on Future Follow-up (r = 0.23) and information on Functional Level (r = 0.22), and lowest correlations with information on Medications (r = 0.15). Table 3 shows the bivariate correlations between the summated rating scale for Availability of Information and the independent variables. Homecare nurses’ scores on the Availability of Information were significantly and positively correlated with items measuring collaboration with the Allotment Office (r = 0.27) as well as with the GP (r = 0.25). Homecare nurses’ assessments of information were weakly but significantly (*p* < 0.05) correlated with all structural factors characterising the municipality nurses were working in, except for the municipality’s unrestricted revenues.

**Table 3:** Bivariate Pearson correlations of homecare nurses’ ratings on Availability of information and collaboration with individual and municipality characteristics. N ranges between 1301 and 1609

		1	2	3	4	5	6	7	8	9	10	11	12
1	Availability of Information Summated Scale	1											
2	Collaboration with Allotment office	.270^**^	1										
3	Collaboration with GP	.252^**^	.187^**^	1									
4	Nurses' educational attainment	ns	ns	ns	1								
5	Years working at current workplace	.146^**^	ns	.093^**^	.192^**^	1							
6	Management position (0 Yes - 1 No)	-.089^**^	-.099^**^	ns	-.248^**^	-.193^**^	1						
7	Centrality index 0 least central – 1000 most central	-.109^**^	-.065^*^	-.162^**^	-.093^**^	-.175^**^	.069^**^	1					
8	Number of inhabitants municipality	-.134^**^	-.067^**^	-.127^**^	-.088^**^	-.099^**^	ns	.551^**^	1				
9	Number of FTEs	.082^**^	.068^*^	.094^**^	.052^*^	.099^**^	-.062^*^	-.727^**^	-.394^**^				
10	Share population ≥ 80 years	.053^*^	ns	.094^**^	.054^*^	.103^**^	ns	-.669^**^	-.373^**^	.771^**^			
11	Unrestricted revenues in NOK	ns	ns	.087^**^	ns	.101^**^	ns	-.578^**^	.069^**^	.536^**^	.492^**^	1	
12	Share of homecare recipients ≥ 80 years in population 80+	.055^*^	ns	.103^**^	ns	.110^**^	.ns	-.605^**^	-.348^**^	.433^**^	.454^**^	.286^**^	1

*p<0.05; **p< 0.01; Pairwise correlations: N ranges between 1301 and 1609

*ns* correlation is not significant at 5 % level

### Multilevel analyses

Multilevel regression analyses were conducted to provide a more accurate analysis of the associations of homecare nurses’ assessments of Availability of Information with horizontal collaborations and structural characteristics at the municipal level. The first step was to estimate the empty model with a random intercept. The empty model for the summated rating scale for Availability of Information showed that 1.39 of the total variation was at the level of municipality (Table 4 Model 1). This corresponded to an ICC of 0.081, suggesting that 8.1% of the total variation in Availability of Information was at the municipal level. The second step was to model the associations between individual nursing characteristics. Homecare nurses having worked longer at the same workplace, and homecare nurses holding a management position had more positive assessments of Availability of Information (Model 2). In the third step (Model 3a) we included homecare nurses’ assessments of collaboration with the GP and with the Allotment Office. Both items are positively and independently associated with homecare nurses’ assessments of Availability of Information. Further analyses (Model 3b) revealed a random effect of nurses’ assessments of collaboration with the GP. This implies that the association between nurses’ assessments of collaboration and Availability of Information depended upon the municipality they were working in. The covariance between the intercept and slope is however close to zero (0.03) indicating no support for that the association of collaboration with the GP is any stronger (or weaker) in municipalities with low average scores on Availability of Information.

**Table 4:** Multilevel regression models for homecare nurses’ assessments of Availability of Information unstandardized coefficients (ß) and standard errors (SE)

	Model 1 ***Empty model***	Model 2	Model 3a ***Collaboration***	Model 3b ***Random slope***	Model 4 ***Structural factors***
*Fixed effect*	*ß (SE)*	*ß (SE))*	*ß (SE)*	*ß (SE)*	*ß (SE)*
Intercept	25.53 (0.14)	25.51 (0.35)	25.46 (0.34)	25.41 (0.34)	28.97 (1.85)
*Nurse-level variables*					
Years at current workplace		0.32 (0.08)***	0.22 (0.08)**	0.23 (0.08)**	0.21 (0.08)**
Management position (0 = yes/1 = no)		−0.73 (0.31)*	−0.54 (0.30)	− 0.51 (0.30)	− 0.45 (0.31)
Collaboration Allotment office (Mean-centred)			0.96 (0.12)***	0.96 (0.11)***	0.96 (0.11)***
Collaboration GP (Mean-centred)			1.00 (0.13)***	0.88 (0.16)***	0.86 (0.17)***
*Structural characteristics municipality*					
Share population ≥ 80 years					−0.20 (0.18)
Unrestricted revenues in NOK					−0.01 (0.02)
Share of homecare recipients ≥80 years of the 80+ population					−0.02 (0.03)
Number of FTEs per 10,000 inhabitants					0.003 (0.002)
Centrality class 1 (most central) – 6 (least central)					−0.27 (0.19)
Municipality size 1 (smallest)-5 (largest)					−0.47 (0.16)**
*Random effect*	*Variance component*	*Variance component*	*Variance component*	*Variance component*	*Variance component*
Municipal level variance					
Intercept (Var U0_j_)	1.39 (0.43)	1.23 (0.40)	0.67 (0.30)	0.56 (0.29)	0.50 (0.62)
Collaboration GP (VarU1_j_)				1.13 (0.47)	1.09 (0.49)
Covariance (U0_j_U1_j_)				0.03 (0.26)	0.08 (0.28)
Nurse level (residual) variance	15.72 (0.65)	15.36 (0.63)	13.88 (0.59)	13.16 (0.59)	13.31 (0.62)
*Deviance (AIC)*	*7885.04*	*7777.29*	*6953.12*	*6941.51*	*6609.77*

*p < 0.05; ** p < 0.01; ***p < 0.001

In the fourth model (Model 4), we entered structural factors at the level of municipality. None of the single structural factors indicating staffing (i.e. FTEs), revenues or caseload had independent statistically significant associations with Availability of Information. Only municipality size showed a significant and negative association with homecare nurses’ assessments of Availability of Information. Independent of all other explanatory variables in the model, homecare nurses’ ratings of the availability of information were, on average, lower in larger than smaller municipalities.

Nurses individual characteristics (Step 2) contributed relatively little to explained variance at both the individual (3.0%) and the municipal level (2.4%). Collaboration with the GP and the allotment office (Step 3) contributed to explain an additional 12.2% of the variance in Availability of Information at the individual level and an additional 10.0% of the variation between municipalities, illustrating a moderate compositional effect. Adding structural municipal level variables (Step 4) explained an additional 4.1% of the variation between municipalities. Homecare nurses’ assessments of collaboration with the GP and with the allotment office contributed most to explaining variation in perceived availability of information, also at the level of municipality.

## Discussion

The current paper provided an overview of the variation in homecare nurses’ assessments of availability of information in the municipal care setting. Assessments were independently and positively associated with perceived collaboration with the allotment office and with the GP. Positive perceptions of collaboration were associated with higher ratings of the availability of information. In addition, small municipalities had, on average, better informational continuity compared to larger ones.

Our study showed that information on medications was considered most often available in the municipality’s documentation system. Nevertheless, more than half of the homecare nurses did not perceive that this kind of information was “Very often/Always” available. Relatively few reported that information related to the recipient’s long-term care needs, such as psychosocial needs or future follow-up, was available in the documentation system. These findings are in line with the results from a previous Norwegian study among health care personnel in municipal care, which showed that only half of the respondents found it easy to obtain a comprehensive overview through the electronic records of the recipient’s needs, the plans for follow-up, and the services received [39]. The continuing predominance of clinical medical patient information in the municipal documentation system suggests a discrepancy between current practices and national as well as international recommendations [40], which state that “health and social care services should be targeted towards preventing and managing declines in intrinsic capacity and improving functional ability in older adults, rather than supporting a siloed and often disjointed approach to management of individual health conditions” p2 [35]. Knowledge about the patient’s values, preferences, and social context developed through a stable provider-recipient relationship, is equally important for ensuring informational continuity and continuity of care [41]. A relevant discussion concerns the best format in which such information should be transferred, i.e. analogous (written/electronic) or synchronous (oral communication) [25]. In case of the latter, a crucial question remains about how to ensure that no important information is lost in increasingly differentiated and complex care systems.

The current study also highlighted the role of collaborations of homecare nurses with core providers within the municipal care system, notably the allotment office and the GP. Net the effect of collaboration with the GP, there was also an effect of collaboration with the allotment office. This illustrated the importance of taking into account the distinctive and supplementary roles of core providers in the municipal care system in ensuring informational continuity in homecare services to older adults. These findings are in line with conclusions from a recent qualitative study suggesting reinforced collaboration and development of a common outlook between GPs, the allotment office and homecare nurses to ensure informational continuity [7]. The strength of the association between collaboration with the GP and perceived availability of information varied across municipalities. Our findings did not indicate any clear patterns suggesting the relevance of structural municipality-level resources for this association. Yet, the results can be seen in line with more general conclusions from the Organization of Economic Co-operation and Development [42] and other studies [43, 44], which highlight that the GP should be more involved in enabling primary care to work in a more coordinated fashion towards groups of chronically ill people, of whom many are older than 65 years.

The associations with collaboration were independent of homecare nurses’ individual characteristics. The number of years homecare nurses worked at their current workplace explained a small, although statistically significant, proportion of variance in assessments of availability of information. Working longer at the current workplace is likely to reflect a certain degree of continuity of care in itself. Homecare nurses with more years of experience may perceive information as more available as they know their patients well and have memorized much information, so that searching for information in the electronic documentation system may seem redundant [14]. It may also indicate the skill of more experienced homecare nurses to locate patient information in the municipality’s documentation system. Another explanation could be that relatively newly employed homecare nurses may have higher expectations of what should be available in the documentation system and thus are more critical towards availability of information.

Finally, the results from the multilevel analyses illustrate the potential impact of municipal context for homecare nurses’ assessments of informational continuity. A small but significant part of the total variation in assessments (8%) is located at the municipal level. Small municipalities have, on average, better informational continuity compared to larger municipalities. Municipalities with a higher number of inhabitants generally have complex care systems, with multiple care providers and GPs, and multiple professions involved in care trajectories. Thus homecare nurses’ “organisation of work” [21, 22], related to ensuring informational continuity is more demanding in larger than smaller municipalities. Informational continuity is important for patient safety and quality of care irrespective of the municipalities’ number of inhabitants. The effect of municipality size may reflect the aggregate impact of differences in staffing, unrestricted revenues and caseload. None of these structural resources had an independent association with informational continuity in municipal care settings. One explanation for these weak associations may be related to our use of an unbalanced design and a limited number of homecare nurses nested within municipalities. Alternatively, it suggests the importance of lower (local) organisational levels of homecare services within a single municipality.

### Strengths and limitations

The current study is the first nation-wide multilevel study of homecare nurses nested within municipalities and their perceptions of availability of patient information. It provides new information on the relative importance of nurses’ individual characteristics, collaborations within the municipal care setting and the structural municipal context. The inclusion of a relatively large number of homecare nurses across a wide demographic and geographical spectrum strengthens the representativeness of the results. This is important, as information on the exact number of eligible nurses working in municipal care services was not available. We were therefore not able to calculate response rates and assess possible sampling bias.

Another limitation is that the use of cross-sectional survey data prevents us from drawing any conclusions about directions of causality in the associations between availability of information and collaborations. Our study also included a relatively crude measure of availability of information that addressed only one aspect of informational continuity: the perceived availability of information in the documentation system of the municipality. Other aspects of informational continuity that address the two-way nature of communication and information exchange are important aspects for further research. The current analysis makes a useful frame for further qualitative and intervention studies to gain new knowledge on the role of horizontal collaborations and municipal-level structures in ensuring informational continuity in homecare services to older adults.

## Conclusions

Municipal documentation systems have a limited focus on the long-term care needs of older care recipients beyond clinical and medical information. Homecare nurses who perceive better collaboration with the allotment office and with the GP, have more positive perceptions of the availability of information in the documentation system. This suggests a potential for facilitating enhanced communication- and care-pathways between GPs, the allotment office and homecare nurses to ensure informational continuity. Multidisciplinary team structures in primary care, supportive information systems and interactive technologies are examples of interventions that enable municipal long-term care services to work in a coordinated manner for older adults in home settings. Closer investigation of informational continuity and horizontal collaboration is required. This can contribute to support the coordinating role of homecare nurses in ensuring informational continuity for older adults with prolonged and complex care trajectories and help develop the facilitating role of (electronic) documentation systems.

## Data Availability

The datasets generated and analysed during the current study are not publicly available since they contain information (i.e. municipality) that could compromise research participant confidentiality. The data are stored on a secure server at Oslo Metropolitan University. Access to the data has been granted to the project’s investigators for a designated period, after which anonymised data will be made available for research purposes through the archives at NSD.

## Abbreviations

GP: Primary physician/General Practitioner
NNO: Norwegian Nurses Organisation
NSD: Norwegian Centre for Research Data
KOSTRA: Administrative database for Municipality-State-Reporting
FTEs: Full-time equivalents
NOK: Norwegian Crowns
ICC: Intraclass correlation coefficient
*AIC*: The Akaike information criterion
SD: Standard Deviation
OECD: Organization of Economic Co-operation and Development
